# The Impact of Spousal Caregiving on Financial Well-Being: An Analysis of Subjective Financial Strain

**DOI:** 10.1093/geroni/igaf122.2358

**Published:** 2025-12-31

**Authors:** Susanna Mage, Michael Hurlburt, Yujun Zhu, Kathleen Wilber, Donna Benton, Kylie Meyer

**Affiliations:** University of Southern California Leonard Davis School of Gerontology, Los Angeles, California, United States; University of Southern California Suzanne Dworak-Peck School of Social Work, Los Angeles, California, United States; Department of Family Medicine, Keck School of Medicine of USC, Los Angeles, California, United States; University of Southern California, Los Angeles, California, United States; University of Southern California Leonard Davis School of Gerontology, Los Angeles, California, United States; Case Western Reserve University, Cleveland, Ohio, United States

## Abstract

Caregiving for a spouse or partner often imposes a significant financial burden, straining household resources and affecting overall financial well-being. This study leverages data from the Health and Retirement Study (2012-2018) to examine the relationship between spousal caregiving and subjective financial strain, focusing on the ability to pay monthly expenses and financial satisfaction. Descriptive findings from regression analyses indicate that a significantly higher proportion of caregivers reported difficulty paying bills (39% vs. 29%, p < 0.001) and dissatisfaction with their financial situation (24% vs. 19%, p = 0.008). Results from population-averaged logistic regression models showed that caregivers had 26% lower odds of reporting the ability to meet their monthly expenses than non-caregivers (OR = 0.74, 95% CI [0.61, 0.89], p < 0.001). Although caregivers had 12% lower odds of reporting financial satisfaction than non-caregivers, this association was not statistically significant (OR = 0.88, 95% CI [0.69, 1.12], p = 0.151), suggesting possible adaptation or coping mechanisms. These findings highlight the financial vulnerabilities of spousal caregivers and underscore the need for targeted policies and interventions to alleviate their economic burdens.

